# Promiscuous potato: elucidating genetic identity and the complex genetic relationships of a cultivated potato germplasm collection

**DOI:** 10.3389/fpls.2024.1341788

**Published:** 2024-07-01

**Authors:** Noelle L. Anglin, Oswaldo Chavez, Julian Soto - Torres, Rene Gomez, Ana Panta, Rainer Vollmer, Marisol Durand, Charo Meza, Vania Azevedo, Norma C. Manrique - Carpintero, Philip Kauth, Joesph J. Coombs, David S. Douches, David Ellis

**Affiliations:** ^1^ International Potato Center (CIP), Lima, Peru; ^2^ Seed Savers - Preservation Department, United States Department of Agriculture Agriculture Research Service (USDA ARS) Small Grains and Potato Germplasm Research, Aberdeen, ID, United States; ^3^ Seed Savers Exchange, Decorah, IA, United States; ^4^ REAP Food Group, Madison, WI, United States; ^5^ Department of Plant Soil and Microbial Sciences, Michigan State University (MSU), East Lansing, MI, United States

**Keywords:** SolCAP SNP array, *in vitro*, diversity analysis, introgression, *Solanaceae*, genetic analysis, genetic resources

## Abstract

A total of 3,860 accessions from the global *in trust* clonal potato germplasm collection w3ere genotyped with the Illumina Infinium SolCAP V2 12K potato SNP array to evaluate genetic diversity and population structure within the potato germplasm collection. Diploid, triploid, tetraploid, and pentaploid accessions were included representing the cultivated potato taxa. Heterozygosity ranged from 9.7% to 66.6% increasing with ploidy level with an average heterozygosity of 33.5%. Identity, relatedness, and ancestry were evaluated using hierarchal clustering and model-based Bayesian admixture analyses. Errors in genetic identity were revealed in a side-by-side comparison of *in vitro* clonal material with the original mother plants revealing mistakes putatively occurring during decades of processing and handling. A phylogeny was constructed to evaluate inter- and intraspecific relationships which together with a STRUCTURE analysis supported both commonly used treatments of potato taxonomy. Accessions generally clustered based on taxonomic and ploidy classifications with some exceptions but did not consistently cluster by geographic origin. STRUCTURE analysis identified putative hybrids and suggested six genetic clusters in the cultivated potato collection with extensive gene flow occurring among the potato populations, implying most populations readily shared alleles and that introgression is common in potato. *Solanum tuberosum subsp. andigena* (ADG) and *S. curtilobum* (CUR) displayed significant admixture. ADG likely has extensive admixture due to its broad geographic distribution. *Solanum phureja* (PHU), *Solanum chaucha* (CHA)/*Solanum stenotomum subsp. stenotomum* (STN), and *Solanum tuberosum subsp. tuberosum* (TBR) populations had less admixture from an accession/population perspective relative to the species evaluated. A core and mini core subset from the genebank material was also constructed. SNP genotyping was also carried out on 745 accessions from the Seed Savers potato collection which confirmed no genetic duplication between the two potato collections, suggesting that the collections hold very different genetic resources of potato. The Infinium SNP Potato Array is a powerful tool that can provide diversity assessments, fingerprint genebank accessions for quality management programs, use in research and breeding, and provide insights into the complex genetic structure and hybrid origin of the diversity present in potato genetic resource collections.

## Introduction

Potato was domesticated approximately 10,000 years ago in the Andes and landraces farmed today still have a wide variety of shapes, skin, and tuber colors that are often not seen in modern varieties (Ovchinnikova et al., 2011). Indigenous potato farmers in the Andes often plant 10–30 different landraces of multiple ploidy levels in the same field (Huaman and Spooner, 2002) to ensure production of at least some tubers annually due to seasonal variation in productivity of individual landraces. This risk mitigation strategy helps protect small holder farmers from new and existing biotic and abiotic stresses in potato production in the Andes. Most commercial cultivars in the USA and Europe are autotetraploids (2n = 4x = 48); however, diploids, triploids, and pentaploids are also commonly found among the cultivated native potato landraces regularly farmed throughout the Andes. Some of the challenges in improving cultivated potato results from a high level of heterozygosity, polyploid genetics, adaptation of native landraces to a short-day photoperiod, complex polysomic inheritance, inbreeding depression, narrow genetic base, and biotic and abiotic factors (Simko et al., 2006; Hamilton et al., 2011; Hirsch et al., 2013).

Markers have been used in many crop plants to assess genetic diversity, determine population structure, discover, and track quantitative trait loci (QTL’s), produce genetic linkage maps, assist in selection for particular traits, understand the influence of genotypes on phenotypes and more - all to understand or improve genetics and key traits. Many different types of molecular markers have been employed since the 1980’s, but single nucleotide polymorphisms (SNPs) are increasingly used due to recent advances in genome sequencing technology and the abundance of SNPs in most crop plants. The affordable cost and high throughput nature of SNP markers have made them powerful tools for genetic analysis of plant species and highly useful in breeding (Bertioli et al., 2014). Discovery of SNPs in simple genomes is relatively easy, requiring collection and evaluation of sequence data; although, in complex genomes such as potato, SNP detection is more difficult due to repetitive segments of the genome and multiple ploidy levels (Mammadov et al., 2012). Genome complexity reduction methods have been developed to aid in the discovery of novel SNPs; nevertheless, it is often challenging to identify SNP markers in polyploids such as potato, tobacco, cotton, canola, and wheat (Mammadov et al., 2012; Bertioli et al., 2014; Logan-Young et al., 2015) due to separating allelic versus homoeologous SNPs which increase the rate of false positives (Clevenger and Ozias-Akins, 2015).

The Illumina Infinium SolCAP 8,303 V1 Potato Array SNPs were originally selected from 69,011 high quality SNPs derived from six commercial potato cultivars ‘Atlantic,’ ‘Premier Russet,’ ‘Snowden,’ ‘Bintje,’ ‘Kennebec,’ and ‘Shepody’ (Hamilton et al., 2011). The Illumina Infinium SolCAP Potato Array V2 contains 12,720 SNPs, including the SNPs from the original V1 (8,303 SNPs) Potato Array with additional markers derived from the Infinium High Confidence SNPs Array (69K, Hamilton et al., 2011), which were selected for genome coverage, candidate genes, and regions with resistance genes. Both potato SNP arrays (V1 and V2) have been used in numerous studies as a genomic tool to improve cultivated potato or gain insight on genetic attributes. These SNP markers were used to measure linkage disequilibrium for genome wide association (GWA) mapping and population structure in European diploid and tetraploid germplasm (Stich et al., 2013). Genotyping a diversity panel of 250 lines of wild species, genetic stocks, and cultivated potato revealed that changes in heterozygosity and allele dosage has not occurred in over 150 years of breeding, but clear selection for alleles in biosynthetic pathways has occurred (Hirsch et al., 2013). The Illumina Infinium SolCAP Potato V1 Array has been used to develop linkage maps (Felcher et al., 2012), genotype populations for QTL analysis (Douches et al., 2014) and assess variation in glycoalkaloid biosynthesis (Manrique-Carpintero et al., 2013, 2014). In other studies, relationships deduced from the SNP markers on the SNP array were generally complementary to existing taxonomic classifications for 74 *Solanum* lines representing 25 wild taxa and were also effective in resolving complex taxa boundaries among germplasm with close genetic relationships (Hardigan et al., 2015).

Conservation of genetic diversity in genebanks is a critical activity, not only for studies of crop diversity, but more importantly for crop improvement in breeding and research. Because breeding efforts and other research using these genetic resources is expensive and time consuming, it is critical that the users of genebank materials receive the genotypic and morphologically correct material they expect. Recent reports have confirmed errors across the plant research and breeding communities of germplasm in transgenic lines, T-DNA lines, cell cultures, and genetic stocks (Bergelson et al., 2016). Genetic contamination or mixing between accessions can occur in any genebank or program handling large numbers of plant material, especially when phenotypic variation is subtle (Bergelson et al., 2016). In the medical field, it is estimated that up to one third of all cell lines may be contaminated or misidentified (Hughes et al., 2007) and although estimates of error in plant genebanks are not thought to be so high, SNP genotyping revealed approximately 5% error in *Arabidopsis* accessions (Anastasio et al., 2011). Girma et al. (2012) evaluated 3,156 accessions of paired yam accessions and found a 20.6% error rate (not true to type) employing 53 morphological descriptors to assess genetic uniformity among paired samples. The entire cultivated sweetpotato collection at CIP was evaluated for genetic identity using 20 SSR markers along with evaluating morphological characterization in the field of paired samples which demonstrated a total of 19.4% error rate in genetic fidelity among paired samples (*in vitro* compared to original mother plants) [Anglin et al., 2021]. Another study evaluated errors in the sweetpotato breeding program at CIP and found a 27.7% error rate which was suggested to have occurred when moving germplasm from *in vitro*, to screenhouse, and then the field (Gemenet et al., 2020). Unfortunately, identifying and sorting out these errors can be extremely difficult and costly, yet it is necessary for genebank operations and the users of a germplasm collection.

The International Potato Center (CIP) in Lima, Peru houses the world’s largest collections of potato. The cultivated potatoes are maintained as clones *in vitro*, in cryopreservation, and distributed worldwide for research, breeding, and education in accordance with the International Treaty for Plant Genetic Resources for Food and Agriculture (ITPGRFA). Internal CIP reports have suggested that identity errors in the *in vitro* germplasm collection have occurred in the past 40+ years of *in vitro* maintenance and regeneration (Perazzo et al., 1999–2000); though, the true extent of these errors within the collection has never been genetically determined. A small subset of the potato collection was previously genotyped using original voucher samples or mother plants and their *in vitro* counterpart and determined an overall error rate of 4.4% in 250 potato accessions (Ellis et al., 2018); however, the entire collection had not been assessed systematically. Further, genetic diversity and population structure of this collection has never been evaluated nor have core or mini core collections been constructed to enhance the utility and access of the genebank collection for users. Therefore, the objectives of this study were to: (i) fingerprint the potato landrace collection in the CIP genebank; (ii) determine if *in vitro* and original clonal mother plants of the same accessions in the collection have identical SNP fingerprints; (iii) evaluate genetic diversity among accessions using SNP markers and assess phylogenetic relatedness; (iv) confirm ploidy levels of material in the collection; (v) reveal population structure among the cultivated taxa; (vi) develop and release a core and mini core collection to represent the majority of the unique genetic diversity in the germplasm collection; (vii) fingerprint potato clones from the Seed Savers Exchange potato collection and compare it to the germplasm collection at CIP to assess overlap of accessions, and (viii) assess if these genetic analyses help to understand the taxonomic challenges existing in the cultivated species in *Solanum* section *Petota*.

## Materials and methods

### Plant material and DNA extractions

**Figure 1:**
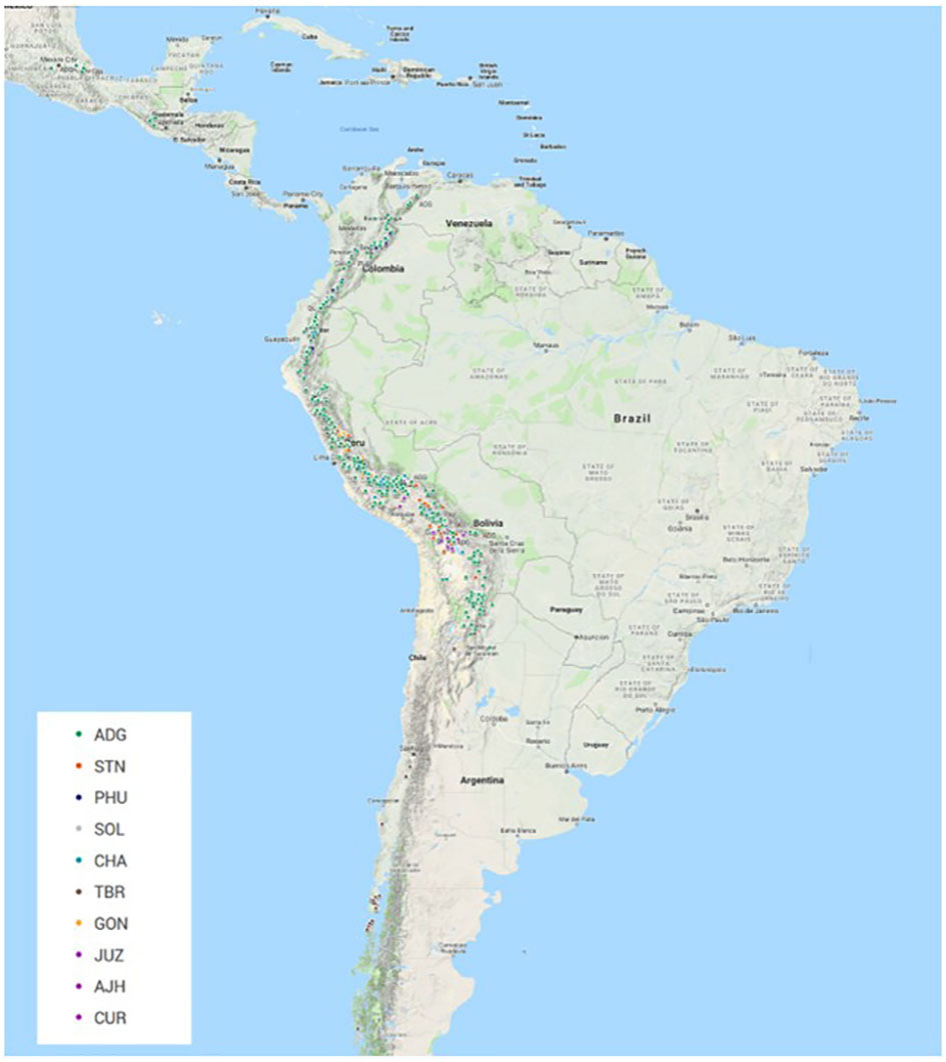
Geographic distribution of 3308 accessions of 3860 that had GIS data from the CIP genebank included in this study. (Not all accessions contain this information in the database and if collected a number of years ago is very difficult to ever acquire). The majority of the accessions were collected or donated from regions in the Andean Mountain range down the West Coast of South America. ADG had the broadest geographical distribution.

All plant material (**Supplementary Table 1**) was obtained from the CIP genebank in Lima, Peru. The accessions in this study consisted of landraces and cultivated improved potato accessions from the genebank collection. Based on existing information and curator knowledge of the species, the accessions that were part of this study included 588 diploids, 191 triploids, 2994 tetraploids, 8 pentaploids, and 79 accessions of unknown ploidy level at the time of this study. The unknown material was acquired from the highlands of Peru and had yet to be classified properly. The number of accessions per taxa (based on Hawkes, 1990, the taxonomic treatment used for potato classifications at CIP) included: 2693 (69.8%) accessions of *Solanum tuberosum* (C. Linneo) subsp. *andigena* (Juz. & Bukasov) [ADG], 120 (3.1%) accessions of *S. chaucha* (Juz & Bukasov) [CHA], 94 (2.4%) accessions of *S. stenotomum* subsp. *goniocalyx* (Juz. & Bukasov) [GON], 27 (0.7%) accessions of *S. juzepezukii* (Bukasov) [JUZ], 190 (4.9%) accessions of *S. phureja* (Juz. & Bukasov) [PHU], 270 (7.0%) accessions of *S. stenotomum* subsp*. stenotomum* (Juz. & Bukasov) [STN], 157 (4.1%) accessions of *S. tuberosum* (C. Linneo) subsp. *tuberosum* [TBR], 14 (0.4%) accessions of *S. x ajanhuiri* (Juz & Bukasov) [AJH], eight (0.2%) accessions of *S. curtilobum* (Juz. & Bukasov) [CUR] and 287 (7.4%) accessions of classified as *Solanum spp* [SOL]. The accessions originated though collecting or donations from Argentina (167), Bangladesh (18), Bolivia (428), Bhutan (5), Chile (126), Colombia (218), Costa Rica (1), Ecuador (305), Guatemala (28), India (1), Mexico (30), New Zealand (4), Peru (2379), Philippines (3), Russia (12), Spain (1), Sweden (8), Venezuela (29), and 97 with no country designation (**Figure 1**). The majority of the collection (97.1%) from this study is derived from collections in South America.

For each accession derived from the CIP genebank collection, DNA was extracted from four *in vitro* plantlets contained in one single test tube and the corresponding original mother plant maintained in the field to evaluate genetic identity among putative clones and to compare identity of the field-maintained samples and *in vitro* samples. The original mother plants have been clonally maintained through the annual regeneration of tubers in the field in the Andean highlands at the CIP field station at Huancayo, Peru (12°01’43”S 75°14’37”W, 3.206 m.a.s.l.). In total, 3860 accessions were used in this study that were determined to be true to type. The accessions found not to be true to type were genotyped but were eliminated from the data set for diversity assessment, phylogeny construction, and STRUCTURE analysis. DNA extractions were performed following a modified CTAB protocol (Doyle and Doyle, 1990). Freeze dried leaf material from 745 potato accessions deposited at the Seed Savers Exchange in Decorah, IA were sent to Michigan State University for DNA extraction. All samples for this study were quantified with a Nanodrop spectrophotometer ND-1000 (Thermo Fisher Scientific Waltham, MA) and run on agarose gels to check quality and quantity of each extract. All DNA samples were diluted to 30 ng/µL for SNP genotyping. SNP genotyping was carried out by Neogene (Lincoln, NE) using the Illumina Infinium SolCAP V2 12K potato SNP array.

### Flow cytometry

Determination of ploidy levels was done by using SNP data to predict ploidy levels (as described in Ellis et al, 2018) and confirmed by flow cytometer as follows; approximately 50–60 mg of young leaf tissue was chopped with a razor blade in a petri dish containing 250 µL of LB01 buffer (15 mM Tris, 2 mM Na_2_EDTA, 0.5 mM spermine.4HCl, 80 mM KCl, 20 mM NaCl, 0.1 % (v/v) Triton X-100, 0.1% β-mercaptoethanol, adjusted to pH 8), [Doležel et al., 1989] to release nuclei (Galbraith et al., 1983). Another 250 µL of LB01 buffer was added and the suspension was incubated for 2 minutes at room temperature. The nuclei suspension was recovered by filtering the cell suspension through a 50 µm CellTrics filter to remove cell fragments and other large debris. Nuclei were stained with 50 µg/mL propidium iodide (PI) and 50 µg/mL RNase was added to the nuclear suspension to prevent staining of double-stranded RNA. Samples were incubated in the dark for two minutes and then analyzed with a BD Accuri™ C6 (BD biosciences, San Jose, CA) Flow Cytometer. The following parameters were used for each sample: Medium Run speed (Fluidics), 1,000 events for the threshold of FL2-H and 10,000 of FSC-H (forward scatter height). The run settings lasted two minutes with at least 400 events for a G0/G1 peak, relative fluorescence intensity of PI-stained nuclei (FL2A and FL3A) and less than 5% of coefficient of variation (CV). Standardization and interpretation of the flow cytometry results were performed using native potato reference standards of known ploidy levels (2x, 3x, and 4x).

### SNP genotyping

Samples were assayed using the Illumina Infinium SolCAP 12K V2 Potato Array on the Illumina iScan (Illumina Inc, San Diego, CA). Samples with greater than 20% missing data were genotyped a second time in an attempt to reduce the amount of missing data. Samples that still showed 20% or more missing data were removed from the data set. The SNP data was analyzed with Illumina’s GenomeStudio (v 1.0) software. Samples were genotyped as diploids (three cluster calling) and as tetraploids (five cluster calling) to generate the specific ploidy phylogenetic trees (i.e. -phylogeny of all diploids); however, analyses of the entire collection was mainly carried out with five cluster calling genotype data since the majority of the accessions are tetraploids. The samples were genotyped as diploids (AA, AB, BB) using the GenomeStudio software auto-clustering feature and the SolCAP custom three cluster calling file (Felcher et al., 2012). The samples were also genotyped with five cluster calling developed for tetraploids (nulliplex=AAAA, simplex=AAAB, duplex=AABB, triplex=ABBB, and quadruplex=BBBB). A total of 5,031 SNPs were obtained from five cluster calling and 8,045 with diploid calling. SNPs that did not produce a clear signal in ≥10% of the individuals or could not be clustered, were removed along with SNPs noted in previous studies to be poor or questionable (http://solcap.msu.edu/potato_infinium.shtml). Genotyping data was deposited in CIP’s dataverse repository and can be found here https://data.cipotato.org/dataset.xhtml?persistentId=doi:10.21223/LBCFCF.

### Data analysis

The sample data set was subsequently filtered to only include putative unique accessions for the evaluation of inter- and intraspecific relationships and to perform STRUCTURE analysis. Principal Components Analysis (PCA) was also employed using the R package ‘adegenet’. The phylogenetic trees were constructed using hierarchical cluster analysis using the unweighted pair group method with arithmetic mean (UPGMA) to calculate the distance matrix in R version 3.2.2 (R Core Team, 2015). The color labels and palettes for the species tree were created using the Dendextend version 1.1.2 and RColorBrewer version 1.1–2 packages in R. iTOL was utilized to annotate and color segments of the tree (Letunic and Bork, 2016).

### Structure

Population structure was estimated using the program STRUCTURE version 2.3.4 (Pritchard et al., 2000; Falush et al., 2003) by assigning the 3,860 accessions to populations or multiple populations based on genotypes produced from 8045 SNP markers. This program infers population structure using a Bayesian approach which identifies clusters and assigns individuals to specific clusters based on Hardy-Weinberg equilibrium and linkage equilibrium. Lambda was initially determined to be 0.5433 for this data set by setting K=1 (the number of populations) and was subsequently set to 0.5433 for the remaining runs. Multiple runs of STRUCTURE were performed by setting K from 1–10. Because STRUCTURE is computationally intensive, the length of burn in and number of MCMC reps was set fairly low to explore the data at different K values and then subsequently increased when an optimal K was discovered. Therefore, the burn-in length was initially 10,000 and MCMC replications were 20,000 for each run (K=2–10). The runs were replicated three times for the initial evaluation. The models employed were admixture and correlated allele frequencies. STRUCTURE Harvester (http://taylor0.biology.ucla.edu/structureHarvester/) was used to determine the appropriate K value (Dent and vonHoldt, 2012) from the results produced. A K=6 was determined to be the optimum. Once the most appropriate K value was determined using a low number of burn-in and replications, the runs were repeated at K =5–7 with an increased burn-in length of 500,000 with MCMC replications of 500,000 to improve the robustness of the Bayesian analysis. Each K value was run in triplicate.

### Predictive taxonomic classification system using STRUCTURE data

The six genetic clusters delineated by STRUCTURE were color coded and the percent of each color was expressed on an accession basis using the color scales rule with conditional formatting in EXCEL. From this, a classification system was derived to describe the frequency of STUCTURE genetic clusters for each species (**Table 1A**). This predictive classification system was tested with known and unknown accessions to determine its utility in the prediction of unclassified accessions into species This classification system was then applied to each accession to see how well it could blindly predict the species determination based solely on morphological data.

Table 1Derivation of a classification system for the prediction of cultivated potato species using the relative % frequency of the six identified STUCTURE populations (represented by six different colors) for each species using the color scales rule with conditional formatting in EXCEL where the relative % frequencies in the cells are color-coded from red (low frequency) to green (high frequency).

**Table:** 

A)							
Species	STRUCTURE population color						
	yellow	red	blue	pink	teal	green
ADG	>10%					>50%
CHA		50–100%			<10%	<30%
GON		>50%			10–40%	NO
AJH		10–50%	NO	20–100%		
JUZ		10–50%	10%	20–100%		NO
PHU	NO		NO		>80%	NO
STN	<20%	>50%	NO	<60%	<50%	<50%
TBR	<10%		>50%	<40%	<5%	<30%
CUR		10–50%	<10%	20–100%		<25%

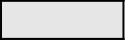
- low to no value of this STRUCTURE population.

NO – zero value for this population.

**Table:** 

B)						
Acc #	yellow	red	blue	pink	teal	green
706032	0	0.565	0	0	0.434	0
706214	0.001	0.704	0	0	0.295	0
706669	0	0.672	0	0	0.327	0
706747	0	0.847	0	0	0.152	0
706831	0	0	0	0	1	0
705211	0.001	0	0.02	0.218	0.003	0.758
705260	0.021	0.002	0.441	0.011	0.004	0.522
705315	0.103	0.002	0	0.007	0.003	0.885
705328	0.068	0.115	0	0.022	0.075	0.72
707848	0.089	0.151	0.071	0.506	0.001	0.182
709027	0.002	0.006	0.792	0.101	0.003	0.096
709028	0.002	0.02	0.802	0.002	0.001	0.173
709029	0.002	0.019	0.801	0.003	0.003	0.172
709031	0.002	0.018	0.807	0.001	0.003	0.168

**Table:** 

C)						
Acc #	yellow	red	blue	pink	teal	green
706032	0	0.565	0	0	0.434	0
706214	0.001	0.704	0	0	0.295	0
706669	0	0.672	0	0	0.327	0
706747	0	0.847	0	0	0.152	0
706831	0	0	0	0	1	0
705211	0.001	0	0.02	0.218	0.003	0.758
705260	0.021	0.002	0.441	0.011	0.004	0.522
705315	0.103	0.002	0	0.007	0.003	0.885
705328	0.068	0.115	0	0.022	0.075	0.72
707848	0.089	0.151	0.071	0.506	0.001	0.182
709027	0.002	0.006	0.792	0.101	0.003	0.096
709028	0.002	0.02	0.802	0.002	0.001	0.173
709029	0.002	0.019	0.801	0.003	0.003	0.172
709031	0.002	0.018	0.807	0.001	0.003	0.168

**Table:** 

D)							
Acc #	yellow	red	blue	pink	teal	green	spp prediction
706032	0	0.565	0	0	0.434	0	gon
706214	0.001	0.704	0	0	0.295	0	gon
706669	0	0.672	0	0	0.327	0	gon
706747	0	0.847	0	0	0.152	0	gon
706831	0	0	0	0	1	0	phu
705211	0.001	0	0.02	0.218	0.003	0.758	adg
705260	0.021	0.002	0.441	0.011	0.004	0.522	adg
705315	0.103	0.002	0	0.007	0.003	0.885	adg
705328	0.068	0.115	0	0.022	0.075	0.72	adg
707848	0.089	0.151	0.071	0.506	0.001	0.182	cur
709027	0.002	0.006	0.792	0.101	0.003	0.096	tbr
709028	0.002	0.02	0.802	0.002	0.001	0.173	tbr
709029	0.002	0.019	0.801	0.003	0.003	0.172	tbr
709031	0.002	0.018	0.807	0.001	0.003	0.168	tbr

A) Classification system to predict the species of cultivated potato based on the frequency of different STRUCTURE populations from accessions with confirmed species designations. B) Example of the raw data from taxonomically undetermined species (X) with relative % frequency of the six STUCTURE populations in EXCEL. C) Example of data from B) expressed with conditional formatting in EXCEL. D) Data from C) with prediction of species based on STRUCTURE populations frequency.

### Core and mini core designation

The software program Core Hunter ver. 3.0.1 (De Beukelaer et al., 2018) in R was utilized to designate a core collection from the SNP data. This program can construct a core collection based on genetic distances calculated from marker data or phenotypic traits. Specific algorithms are employed to optimize selection of entries based on metrics specific of the purposes of a core collection (represent whole diversity, extreme genotypes, or a distribution pattern). In this study, a core collection was built to represent the genetic diversity of the 3860 accessions in the CIP collection using the accessions to nearest entry function (ANE). Five cluster calling from the SNP data (nulliplex=AAAA, simplex=AAAB, duplex=AABB, triplex=ABBB, and quadruplex=BBBB) and species designations were utilized for this analysis. A selection of 11.68% of the total accessions were chosen for representation in a core collection. After Core Hunter produced results, the selections were evaluated, and minor modifications were made based on knowledge of agronomic values, traditional knowledge, and trait information. These minor modifications made by the potato curator were only performed for closely related accessions within a grouping. An ultimate core collection of 451 accessions was produced. The same process was repeated for the selection of the mini core collection resulting in a mini core of 45 accessions.

## Results and discussion

### Genetic parameters and ploidy level

The Illumina Infinium SolCAP V2 12K potato SNP array was employed to evaluate genetic identity, diversity, and population structure of the global *in trust* potato germplasm collection at CIP. The potato germplasm collection at CIP is composed of landraces and native potato material mostly originating from the Andes region of South America. The species classification used in this study is based on the taxonomy of Hawkes (1990). Although Spooner taxonomy (Spooner et al., 2007) is widely used in potato classifications, CIP and other institutions worldwide have historically used the taxonomic treatment of Hawkes’s (Hawkes, 1990) and still employ it to classify potato due to familiarity, history of Hawkes working at CIP, and its utility for classifying genebank materials. A total of 3,308 of these accessions have GIS data in their passport data. A map of the geographic distribution of accessions and species that had GIS data can be seen in **Figure 1** with ADG having the broadest geographic distribution of any of the potato species. The majority of the collection (~95%) that was part of this study was derived from South America which is typically very unique (mostly all short day adapted), compared to North America and European germplasm (long day adapted).

For each accession in this study, DNA was extracted from both a bulk of four *in vitro* plantlets contained in one single test tube and a corresponding original mother plant to evaluate genetic identity among putative clones maintained of the same accession. Genetic identity was determined through genotyping with the Illumina Infinium SolCAP Potato Array (V2) and morphological comparisons of the mother plant with an *in vitro* plantlet of the same accession was done in the field to ensure basic plant traits (descriptors) were equivalent among the pair. Only material determined to be true-to-type (TTT) by SNP fingerprinting (*in vitro* and mother plant matched in genotype) were used for analysis in this study as there were reports of identity errors in the CIP collection (Perazzo et al., 1999–2000; Ellis et al., 2018; Anglin et al., 2021) as well as errors being reported in other plant collections (Anastasio et al., 2011; Girma et al., 2012; Gemenet et al., 2020). The genotyping data demonstrated an error rate of 19.9% between the matching pairs of clones. In total, 3,860 accessions had matching fingerprints for a mother plant with their *in vitro* counterparts and matched morphologically, and thus, these accessions were further used in this study to evaluate genetic diversity and population structure. The remaining accessions that contained errors (did not have matching genotypes between mother plant and *in vitro* pairs) were not included in this study and will be evaluated further to correct the errors in the collection prior to being distributed out to users or utilized for research purposes.

The five cluster SNP data was applied to predict ploidy levels of all the accessions. Previously in Ellis et al. (2018), SNP data was utilized to predict ploidy and subsequently confirmed with flow cytometry, and this method was also repeated here, since species classification in potato is not always an adequate indicator of ploidy level (Ghislain et al., 2006; Ellis et al., 2018). In both studies, SNP data was found to be an accurate and fast indicator of ploidy levels. Many of the accessions in the collection were originally assumed to be a particular ploidy solely based on their species classification or morphological characterization and never evaluated further. Thus, the SNP data were utilized to confirm ploidy level of each accession since this is an important trait for breeding and management of the collection. Overall, 92.5% of the potato accessions had ploidy data that matched historical data based mostly on morphological and species designation and 7.45% (288 accessions) needed adjustments. Of those, seventy-seven accessions (of 288) were not yet classified to a species level, and thus, had no ploidy data collected until the SNP data was applied. These data were confirmed with flow cytometry and the predicted ploidy based on SNP data matched the flow cytometry data. Where ploidy levels did not match the original ploidy determinations, flow cytometry was used to confirm the ploidy levels and, in all cases, the predicted ploidy based on SNP was confirmed with the flow cytometry data. Importantly, based on these data, most potato species had a mix of various ploidy levels with no species being purely one single ploidy. For example, AJH was mostly diploid (79%), yet some AJH accessions were determined to be either triploid (14%) or tetraploid (7%). PHU was similar in that most of the PHU accessions were diploid (87%), but a few accessions were triploid (6%) and tetraploid (7%). STN was mostly diploid (74%); however, some triploids (8%) and tetraploids (18%) were revealed. All of the species had some minor variation of ploidy levels within the species; however, some were only variable for one accession or very few accessions. Some of these observations could be due to incorrect species classification of an accession, from mixed ploidy within a species, or both classification and mixed ploidy (**Supplementary Table 1**).

**Figure 2:**
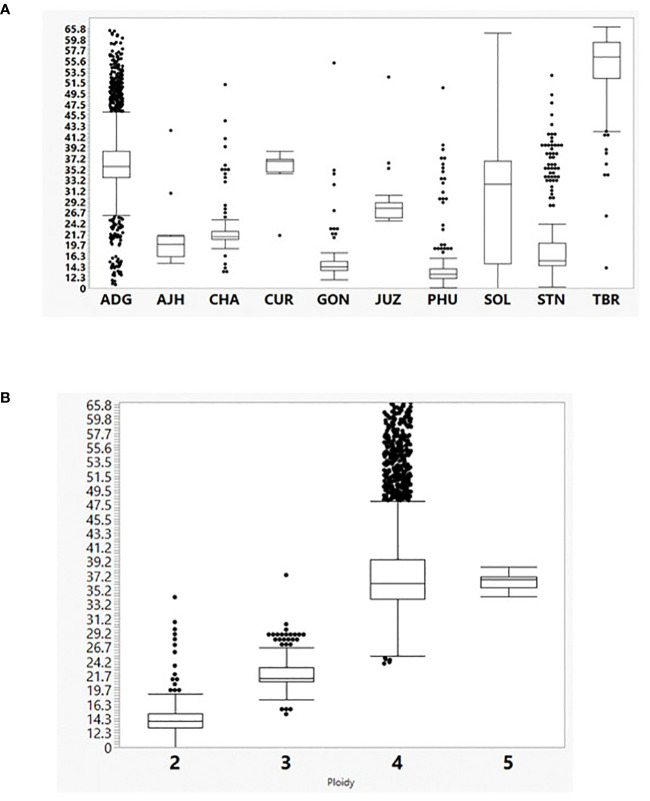
**(A)** The percent heterozygosity (y axis) of the 3860 cultivated potato CIP genebank accessions used in this study grouped by species classification (x axis). Hawkes, 1990: *Solanum tuberosum* (C. Linneo) *subsp. andigena* (Juz. & Bukasov) [ADG], *S. chaucha* (Juz & Bukasov) [CHA], *S. stenotomum* subsp. *goniocalyx* (Juz. & Bukasov) [GON], *S. juzepezukii* (Bukasov) [JUZ], *S. phureja* (Juz. & Bukasov) [PHU], *S. stenotomum* subsp*. stenotomum* (Juz. & Bukasov) [STN], *S. tuberosum* (C. Linneo) subsp. *Tuberosum* [TBR]*, S. x ajanhuiri* (Juz & Bukasov) [AJH], *S. curtilobum* (Juz. & Bukasov) [CUR] and unclassified accessions as *Solanum spp* [SOL]. **(B)** Percent heterozygosity (y-axis) grouped by ploidy level based on SNP data.

The SNP data were also utilized to calculate heterozygosity. The heterozygosity in the entire potato collection ranged from 9.7% to 66.6% with an average heterozygosity of 33.5% (**Figure 2**). The TBR accessions were the most heterozygous species in the collection which was also seen in a previous study evaluating a subset of the CIP potato collection (Ellis et al., 2018). TBR was more heterozygous than even the pentaploid accessions (CUR). There are, however, very few CUR accessions (eight) within the germplasm collection compared to 157 accessions of TBR which could have lowered the heterozygosity in CUR. The ADG accessions were the most variable species with a wide range of heterozygosity (10.8 – 62.8%) along with the accessions only classified as *Solanum* (SOL) which included diploid, triploid, and tetraploid accessions. Overall, heterozygosity tended to increase with ploidy level where species generally thought to be diploids had lower heterozygosity than species with higher ploidy levels (3x, 4x, and 5x) with heterozygosity maximized with some of the tetraploids.

### PCA

**Figure 3:**
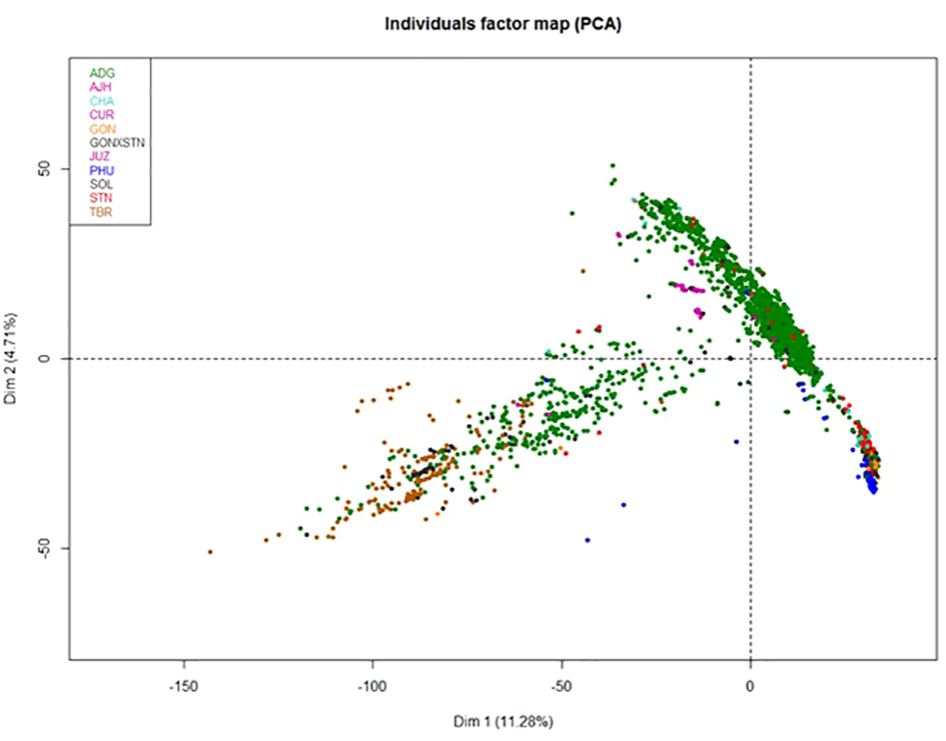
Principle Component Analysis (PCA) of the 3860 potato accessions from the genebank genotyped by the SolCap SNP Array.

Because of the size of this data set, the 3,860 true to type (TTT) accessions were analyzed by Principal Components Analysis (PCA) to help reduce the dimensionality of the data, yet still preserve much of the variability (**Figure 3**). The first principal component contained 11.28% of the genetic variance and the second component contained 4.71% of the variance. Many of the species in the PCA were overlapping suggesting they are highly correlated. This suggests significant introgressions or hybridization has occurred among these species and accessions or the genus is over described. The ADG species shown in green, split into two distinct groupings, one of which overlapped slightly with TBR. This was also observed in a separate study evaluating the Colombian Central Collection of potatoes (Manrique-Carpintero et al., 2023). The other portion of ADG overlaps with most of the other cultivated potato species. The STN, GON, and CHA species also appear to be highly correlated with PHU and some of the accessions only classified as *Solanum* species.

### Phylogeny

**Figure 4:**
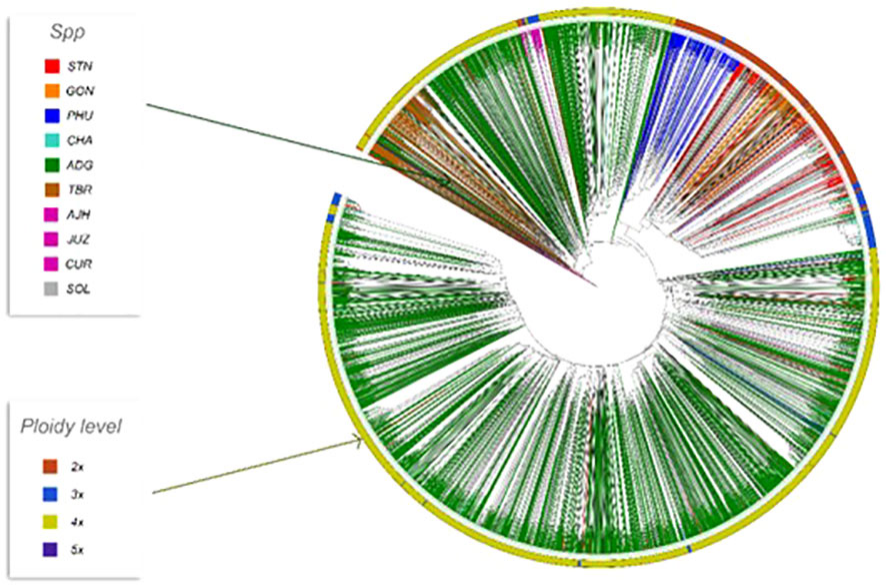
Dendrogram based on the data from the Illumina Infinium SolCAP V2 12K potato SNP array of the 3860 accessions of cultivated potato germplasm from the genebank used in this study. The outside ring is ploidy level. The branches are colored by species designation in the CIP database.

Phylogenetic trees were constructed for the entire set of true-to-type cultivated genebank accessions (3,860 accs) to look at inter- and intraspecific relationships on a collection wide basis (**Figure 4**). Generally, species and accessions in the entire collection (3,860 accs) of different ploidy levels clustered as in a previous study (Ellis et al., 2018) which assessed genetic variability of a limited number of material but included representative accessions of the cultivated potato species from the CIP genebank. The *S. x ajanhuiri* (AJH)*, S. curtilobum* (CUR)*, S. x juzepeczukii* (JUZ) accessions, which are considered bitter potatoes, grouped together even though they consist of diploids, pentaploids, and triploids, respectively. *S. tuberosum* subsp*. andigena* accessions (ADG) which represented 69.8% of the true-to-type cultivated collection, split into different clades, one of which was more similar to bitter potato accessions (AJH, CUR, JUZ) and TBR, with the other clade clustering on its own. *S. stenotomum* subsp*. goniocalyx* (GON) and *S. stenotomum* subsp*. stenotomum* (STN) were intermixed as was seen previously (Ellis et al., 2018) and did not cluster monophyletically. Hawkes (1990) classified these as subspecies suggesting that STN and GON did not have significant morphological differences and are highly related. Spooner et al. (2007) previously collapsed these subspecies (STN and GON) along with other potato species (PHU, ADG, and CHA) into a single group: *S. tuberosum* Andigenum group. The results here support Spooner taxonomy with the lumping of STN and GON into a single taxonomic group.

**Figure 5:**
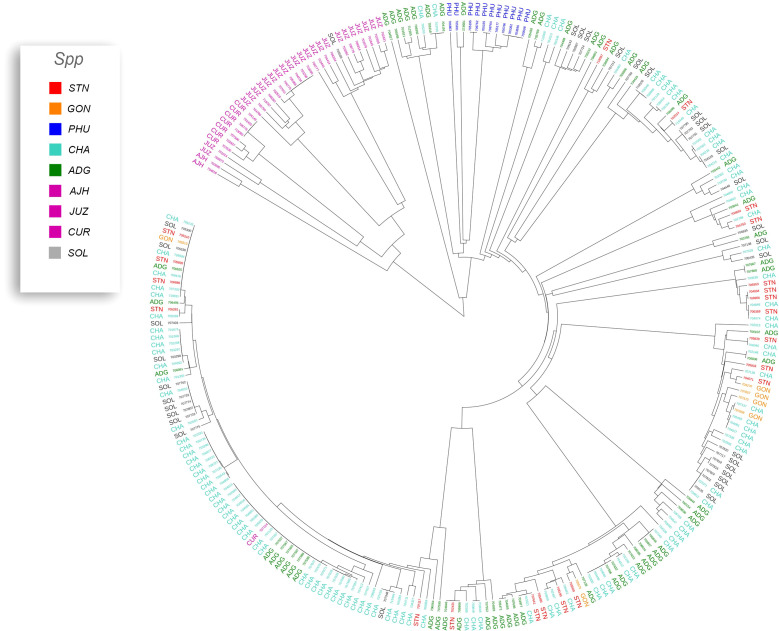
Dendrogram of all diploids in the cultivated potato collection.

Previous studies have shown that species designation is not a good indicator of ploidy level in potato (Ghislain et al., 2006; Spooner et al., 2007; Ovchinnikova et al., 2011; Ellis et al., 2018). Therefore, all accessions that had a profile consistent with a diploid pattern from the SNP data were grouped together and a phylogeny was constructed (**Figure 5**) to assess interspecific relationships among diploid potatoes. In general, this included accessions from *S. phureja* (PHU)*, S. stenotomum* subsp*. goniocalyx* (GON)*, S. stenotomum* subsp*. stenotomum* (STN)*, S. ajanhuiri* (AJH), accessions only classified as *Solanum*, and a few miscellaneous accessions from other taxa that appeared to be diploid. The majority of *S. phureja* (PHU) accessions clustered together. A few exceptions are noted, and it is possible these are either hybrids or the species designation of these accessions are misclassified. Accessions classified as *S. ajanhuiri* (AJH) also clustered together. Both Spooner et al. (2007) and Hawkes (1990) considered AJH as a distinct potato species. Additionally, a few genetic redundancies were apparent in the tree among these diploid accessions.

**Figure 6:**
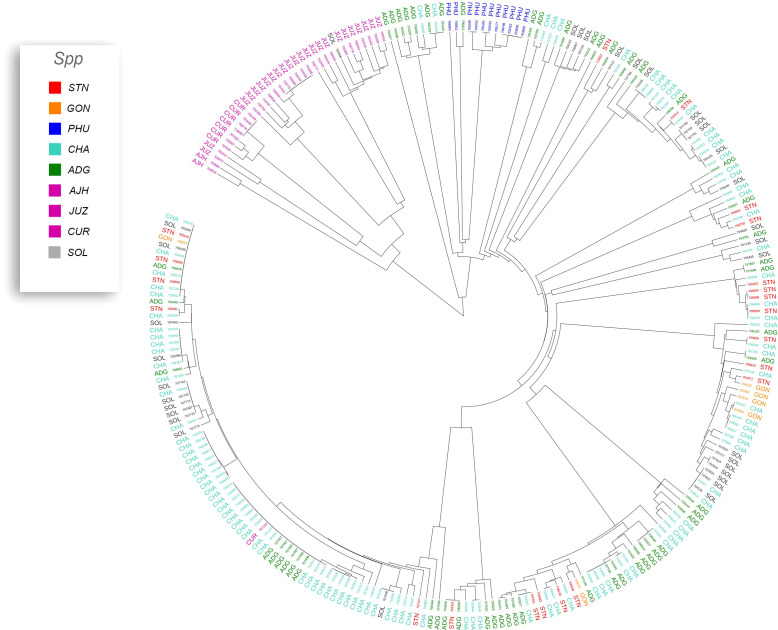
Dendrogram of triploids and pentaploids in the cultivated potato collection.

A phylogeny of the triploids and pentaploids was also constructed (**Figure 6**) to examine intra- and interspecific relationships and none of the taxa formed a distinct monophyletic clade. Overall, the majority of the triploid accessions (JUZ and CHA) displayed little genetic variability with short branch lengths between the various accessions suggesting high genetic similarity. Since triploids are generally an evolutionary dead end, it is reasonable to believe that their variability would be low. There were eight pentaploids included in the analysis of which four accessions were identical based on the SNP data suggesting they are likely genetic duplicates. The majority of the accessions of bitter potatoes (AJH, CUR, and JUZ) with one exception (CIP 707124 CUR) all clustered together. All the species of bitter potatoes were considered as unique by both Hawkes (1990) and Spooner et al. (2007). *S. chaucha* (CHA) also had many genetic similarities among accessions shown by short branch lengths between individuals which supports the suggestion above that triploids have low genetic variability. Some of the unclassified *Solanum species* accessions were genetically similar, based on the SNP analysis to other classified accessions suggesting they are redundant. Further, some potato accessions classified as other species also grouped within some of these non-variable regions in the phylogeny. This data suggests either that SNPs from the array may not be sufficiently dense enough in the genome to genetically distinguish these accessions as different species or there is very little genetic variability in the accessions that are triploid and pentaploid. It is also possible that ascertainment bias from the SNP array is the cause of this due to the lack of rare variants. Another possibility is that these accessions were duplicates or closely related which was not apparent when they were originally introduced into the collection. One potential drawback of utilization of SNP arrays on diverse material is that SNP arrays can lead to ascertainment bias especially when the markers on an array were discovered from a small number of samples or samples that do not represent the broader population (Caruana et al., 2019). Arrays can lack a significant proportion of rare variants and be biased towards variants in the populations used to develop the respective array (Geibel et al., 2021).

### Structure

**Figure 7:**
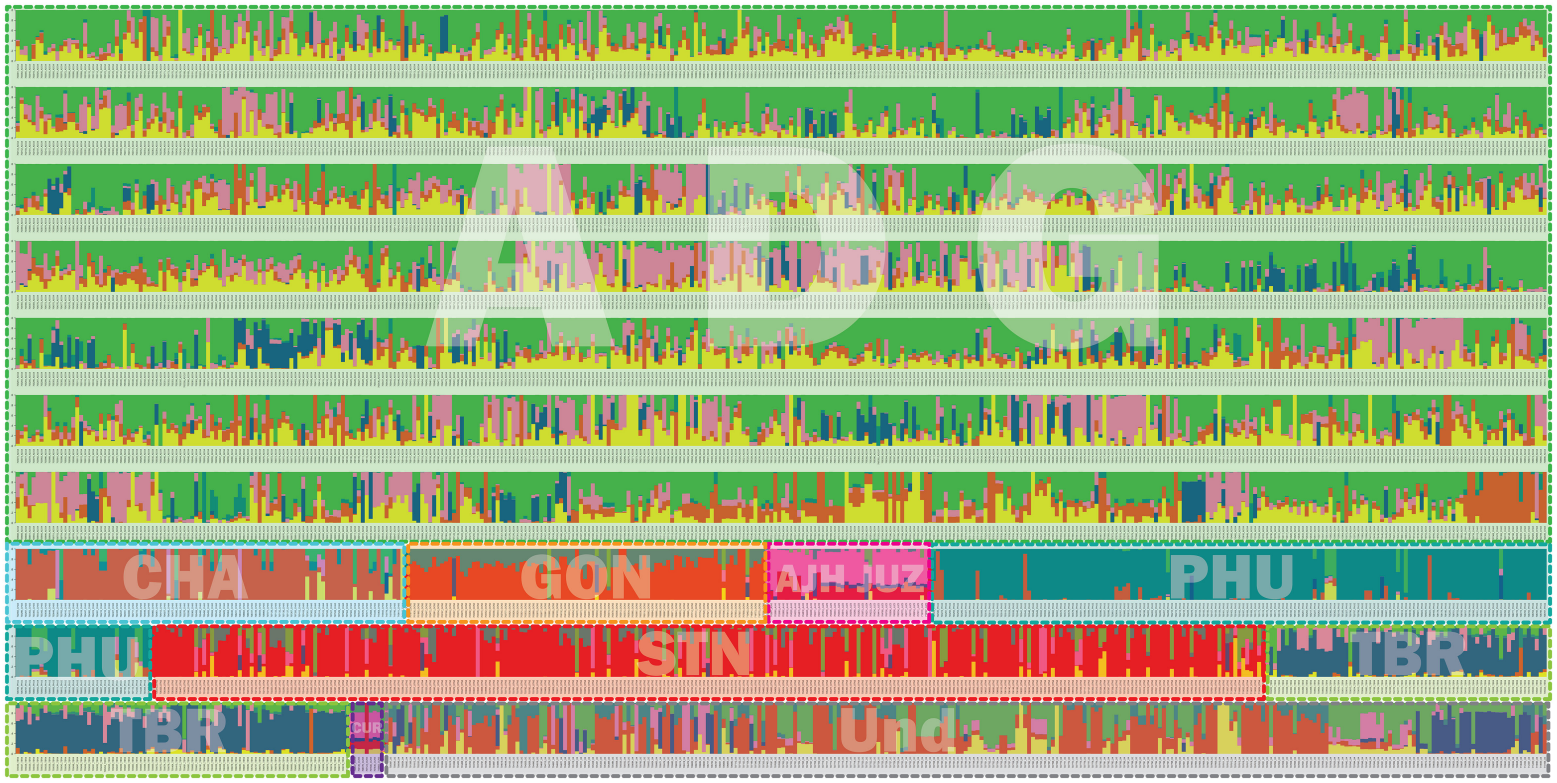
Structure data for 3860 cultivated potato accessions from the CIP genebank used in this study. The species are represented by 3 letter codes with “ND” signifying not determined as they were new introductions and not fully classified at the time of this work. The accessions are classified per taxa based on Hawkes, 1990 which has long been the only taxonomic system for potato classifications at long been the only taxonomic system for potato classifications at CIP and included: *Solanum tuberosum* (C. Linneo) *subsp. andigena* (Juz. & Bukasov) [ADG], *S. chaucha* (Juz & Bukasov) [CHA], *S. stenotomum* subsp. *goniocalyx* (Juz. & Bukasov) [GON], *S. juzepezukii* (Bukasov) [JUZ], *S. phureja* (Juz. & Bukasov) [PHU], *S. stenotomum* subsp*. stenotomum* (Juz. & Bukasov) [STN], *S. tuberosum* (C. Linneo) subsp. *tuberosum* [TBR]*, S. x ajanhuiri* (Juz & Bukasov) [AJH], *S. curtilobum* (Juz. & Bukasov) [CUR] and unclassified accessions as *Solanum spp* [ND]. The six populations are represented by colors = green, pink, yellow, red, dark blue, and turquoise.

Population structure analysis allows for understanding of gene flow, admixture, and inference of demographic histories of populations (Stift et al., 2019). Simulated data has demonstrated that the program STRUCTURE is more robust than other methods in handling biases due to mixed ploidy levels (Stift et al., 2019) and because this data had a mixture of diploid, triploid, tetraploid, and pentaploid accessions, STRUCTURE was employed to provide a robust genetic structure analysis and understand gene flow and admixture of a large pool of potato landrace germplasm. The genotyping data was used in the program STRUCTURE to determine the number of genetic clusters in the genebank collection. A total of six genetic clusters were observed (**Figure 7**). One striking feature is the high level of admixture and substantial gene flow observed among the majority of accessions genotyped suggesting and supporting the notion of high heterogeneity of potato. The extensive gene flow was especially apparent in the ADG accessions which appeared to pick up alleles from every lineage and contained significant introgressions within most accessions. Hoopes et al. (2022) using whole genome sequence (WGS) data recently reported significant allelic diversity in cultivated tetraploid material from Europe and North America demonstrating ancestral introgressions from wild species that predated breeding efforts. All the cultivated species from South America were included in this study and many of these could be hybrids, with either wild species or rare cultivated material not in the genebank collection in their background as some of these introgressions do not appear to be derived from the cultivated species included in this work. More genotyping of wild potatoes would be required to determine the origin of putative wild alleles as they do not appear to be completely derived from the cultivated collection.

The phylogeny of the ADG accessions split into two major groups and the STRUCTURE data suggests that one of these genetic groupings had more alleles shared with the bitter potatoes represented in pink (AJH, CUR, and JUZ) which explains why a subset of the ADG accessions clustered in the phylogenetic tree with the bitter potatoes and TBR, which also contains this introgression. After closer morphological examination, these ADG accessions with this introgression, have characteristics more similar to the wild potato species and tend to be more bitter to the taste than the other ADG accessions, suggesting higher glycoalkaloid level in these tubers than the remaining ADG accessions which do not contain the putative bitter alleles (pink). Spooner et al. (2007) and Hawkes (1990) both recognized AJH, CUR, and JUZ as distinct species. Yet, it is interesting that the bitter potatoes despite being different ploidies, and different species according to both Spooner and Hawkes, cluster together in the phylogeny (**Figure 4**) and share fairly similar introgressions\population structure giving the appearance they are genetically similar or related. Hawkes (1990) proposed that CUR arose as a hybridization from JUZ and ADG. The STRUCTURE data supports this showing CUR having shared alleles with the bitter potatoes (pink) and ADG (green and yellow). Hawkes (1990) also proposed that AJH arose from a hybridization among STN x *S. megistacrolobum* (wild species) and that JUZ was a hybrid of STN x *S. acuale* (wild). While there were no wild species included in this dataset, both AJH and JUZ show signatures of introgression from the CHA/STN population (red) partially supporting Hawkes’s hypothesis. More data would be required from the wild species to evaluate these hybridization theories of Hawkes; however, the cultivated species proposed by Hawkes are supported by these data.

The STRUCTURE data suggests that GON is a hybrid of PHU and the CHA/STN lineage with a larger contribution of the alleles from CHA/STN lineage than the PHU lineage. These results support Spooner taxonomy (2007) which eliminated GON as a separate species/subspecies and lumped it together within the *S. tuberosum* Andigenum group. However, Spooner also lumped CHA and STN into the *S. tuberosum* Andigenum group, whereas Hawkes kept these as separate species or subspecies. Hawkes proposed GON as a species that diverged from STN, and these data show the divergence is likely due to an introgression from the PHU population. PHU and GON do have an overlapping geographical distribution with PHU ranging from Venezuela to central Bolivia and GON found from Northern Peru to central Bolivia (**Figure 1**). The STRUCTURE and phylogenetic data partially support Spooner taxonomy in that CHA and STN appear to be derived from a single population and are not unique. In contrast, PHU does appear to be derived from a unique population; however, PHU was lumped by Spooner into the *S. tuberosum* Andigenum group. Furthermore, CHA and STN appear to share alleles from the same population also partially supporting Spooner taxonomy of lumping these together into the *S. tuberosum* Andigenum group.

Three of the six genetic clusters identified in STRUCTURE (PHU, CHA/STN, and TBR) had lower introgressions overall, with the remaining genetic clusters arising through extensive introgression/hybridization, which appears to be a continuous process in potato. These results suggest and confirm the promiscuity of potato in picking up and exchanging alleles whenever and wherever possible. Several of the accessions classified as TBR, the most cultivated potato species in USA and Europe, appear to have accessions displaying significant introgressions from the bitter potatoes represented with the pink color (CUR/JUZ/AJH) and some introgression from ADG. Both Spooner and Hawkes recognized TBR as a distinct species which is supported by this analysis as well. Several of the ADG accessions have some introgressions from the TBR population as well, along with other introgressions which may be derived from wild species. The cultivated and wild potato curators noted wild characteristics and more bitterness in tubers of the ADG accessions with the pink coloring in the STRUCTURE (**Figure 7**) supporting the hypothesis that these may be derived from wild species or have significant wild species introgressions. Further, ADG and CUR accessions were the most admixed species in this data set. This is likely due to its wide geographical distribution of ADG relative to the other potato species, which allowed ADG to gain access to other potatoes (possibly wilds) and shared alleles freely producing all the introgressions.

Utilizing the data from the STRUCTURE analysis, a classification system was derived using the percent relative frequency of the six STUCTURE populations for each species (**Table 1**) and then applied to the accessions which were classified as unknown (SOL) or of questionable species determination and for the putative hybrids along with confirmation of species designation performed independently in the field. The accuracy of the classification system varied with species yet proved highly predictable (>75% correct) for species designation of unknowns of ADG (75% correct), GON (76% correct), PHU (100% correct), STN (88% correct) and TBR (100% correct). In contrast, the classification system derived for AJH, CUR, and JUZ, which remain as recognized species by Spooner et al. (2007) were not predictive for the putative species likely due to the low number of accessions tested of these species was 5, 1, 1, respectively. Interestingly, CHA alone also had very low predictability (6% correct); however, all incorrect calls were classified morphologically as STN (50%) or STN hybrids, with which CHA groups in the dendrogram (**Table 1**). Overall, the percent of correct calls confirmed by morphological analysis was 76% with individual pure species calls however, if putative hybrids were included, the predictive values of this classification system increased to 87% if a positive species call was extended to one of the suspected hybrid parents.

These data support Hawkes (1990) taxonomy in that the use of genetic data, SNP data in this case, had predictive value in identifying and differentiating STN, PHU, GON, and TBR. These data also correctly predict ADG although the STRUCTURE data shows a very high level of admixture in ADG, and thus, the derived classification system only relies on two of the six STRUCTURE genetic clusters. Prediction of species in genebank accessions can help fill in major gaps in databases allowing users to better select individual accessions for their research needs. Further, it will help the genebank delineate material as many of the accessions in this study were only classified as *Solanum species*.

This work further demonstrates the utility of complimentary methods phylogenetics, STRUCTURE, PCA, and a classification system produced from SNP genotyping data for reticulate phylogenetic relationships, allowing a new perspective on the genebank collection that was not possible to understand previously from morphological data alone. This is especially applicable to many germplasm collections in which growing out the entire collection and evaluating it in a single year is not feasible. Each of these analyses have different algorithms \ approaches but taken as a whole lead to a robust analysis and insights on the species, hybrid origin of individuals or species, and the overall diversity of the collection. These data revealed accessions that are genetically similar or duplicates which, once verified, can now be archived or eliminated for cost efficiencies, and reduce the high cost of maintaining clonal collections. The data also revealed accessions that were misidentified taxonomically which is important to correct for end users to be able to target the species or accessions they desire. The STRUCTURE data was a helpful tool to identify misclassified accessions in the genebank collection and visualize the extent of admixture within and between accessions of the same genetic lineage. For example, several of the accessions labeled as CHA do not appear to be classified properly as their STRUCTURE data does not fit the pattern of other CHA accessions nor do they fit the predictive classification system derived from the data. CIP 707124 which was originally classified as CUR appears derived 100% from the CHA/STN population in STRUCTURE which explains why this accession clustered with CHA in the phylogeny and is predicted as CHA from the classification system. Another example, CIP 703882 which was classified as GON and thus assumed to be a diploid, yet the SNP data suggested it as a tetraploid, the STRUCTURE data did not appear like other accessions of GON with admixture more similar to ADG, and this accession did not cluster with other GON accessions and is likely ADG. CIP 703545 originally classified as PHU also did not produce a pattern in STRUCTURE similar to the other PHU accessions, nor did it cluster with the PHU accessions and clustered with CIP 703668 STN suggesting it is misclassified as a PHU. The classification system offered two possible designations, either GON or STN, which can be verified in the field. The collection also had 287 accessions that were only identified as SOL and without a potato taxonomist to evaluate them, they have remained unclassified. The data collected here helps parse those accessions into different ploidies and into a species designation which can be easily predicted based on their STRUCTURE admixture along with their clustering in a phylogenetic tree. Indeed, the derivation of a classification system from the STRUCTURE data identified these formally unknowns to species with >75% accuracy based on field morphological determinations. These types of analyses can aid genebanks in determining and classifying taxa and benefit the overall management of the collection.

### Seed Savers

**Figure 8:**
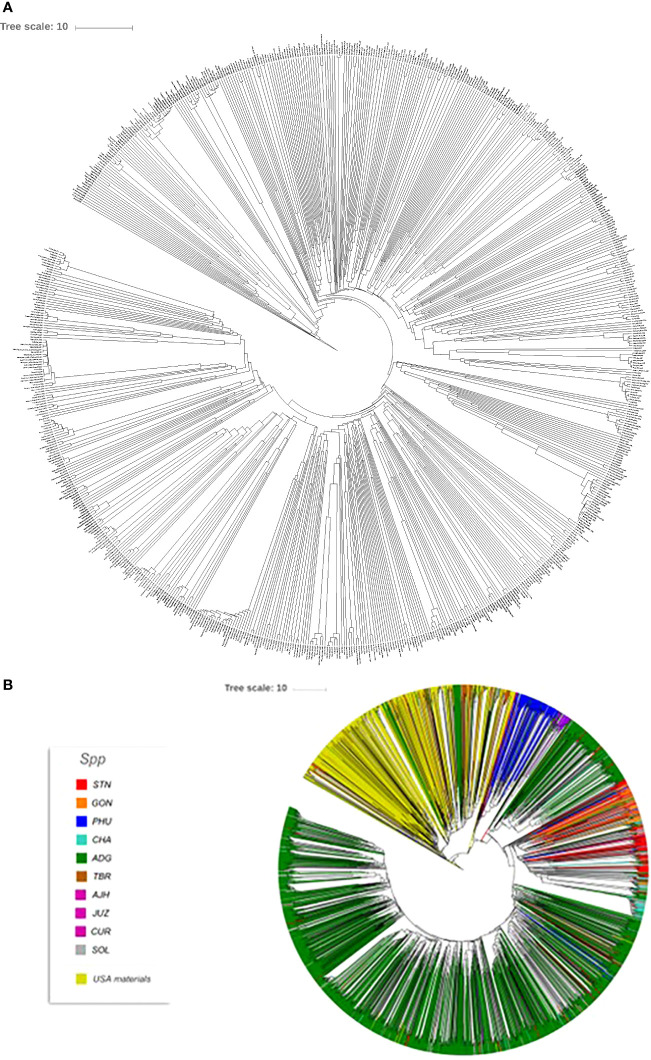
**(A)** Dendrogram of 745 accessions from the Seed Savers collection and **(B)** phylogeny of the Seed Savers (yellow branches) relative to the 3860 cultivated accessions from the CIP collection used in this study.

Seed Savers is an organization that maintains heirloom and historical varieties. Their mission is to collect, regenerate, store, and distribute varieties (https://www.seedsavers.org/mission) and have been operating since 1975. Their mission is analogous to the mission of any genebank. In order to understand the potential overlap in the Seed Savers potato germplasm collection and that of CIP, Seed Savers was contacted, and a portion of their potato collection was also fingerprinted. A total of 745 potato accessions from the Seed Savers collection were genotyped with the Illumnia Infinium SolCap V2 potato SNP Array and a dendrogram was produced showing the variability of the potatoes in the Seed Savers collection (**Figure 8**). Overall, much of the Seed Saver collection consists of unique accessions with divergent fingerprint patterns. Relatively few accessions seemed to be genetically similar to one another indicating that the portion of the collection which was genotyped does not contain a high number of genetic redundancies. Further, when compared to the entire CIP collection, no redundancies were found between the two collections suggesting that these potato collections are quite different resources, each contributing unique diversity for users of the collection. Most of the material from the Seed Saver collection appears to be more related to CIP’s TBR accessions based on a combined phylogeny (**Figure 8**) of the Seed Savers and CIP material which makes sense as the Seed Savers collections are heirloom varieties from North America which have long day adaption and therefore are quite different to the short day adapted germplasm maintained at CIP.

### Core and mini core collection

It is challenging for users of germplasm collections to narrow down the total accessions from an entire collection to a manageable subset for use in research projects. One strategy that has been useful is the development of core and mini core collections from genebank collections (Frankel and Brown, 1984). A core collection is a subset of the entire germplasm collection, typically 10%-15% of the total collection that represents the majority of the genetic diversity in the entire germplasm collection with little redundancy (Brown, 1989). This concept was developed to improve the use of the germplasm materials since genetic resource collections can contain several thousands of accessions which is unwieldy for researchers to mine (Upadhyaya and Ortiz, 2001) and to help researchers find traits of interest in a smaller, more manageable subset. A mini core collection takes this idea one step further with additional reductions to target a sub-collection by sampling the diversity in approximately only 1% of the germplasm collection, so that traits expensive to measure can be assessed in an even smaller subset. This concept has been useful in previous studies to identify germplasm from the larger collection which contain a trait(s) of interest. If a particular trait is found in a core or mini core, a researcher can identify the original grouping of the accessions from the entire collection in the design of the subset, and subsequently track back to that cluster of accessions for identification of more accessions with the trait(s) of interest. Also, because the potato germplasm collection is rather large and, as a clonal crop, is expensive to distribute as phytosanitary clean *in vitro* clones (in comparison to distribution of seed germplasm), frequently clonal genebanks need to greatly limit total germplasm distribution to users. Core and mini core collections aid in this by allowing genebanks to send requestors a smaller collection of diversity represented in the collection to aid breeders and researchers in targeting desired accessions for trait evaluations. A core and mini core collection has not previously been developed for the CIP genebank material.

**Figure 9:**
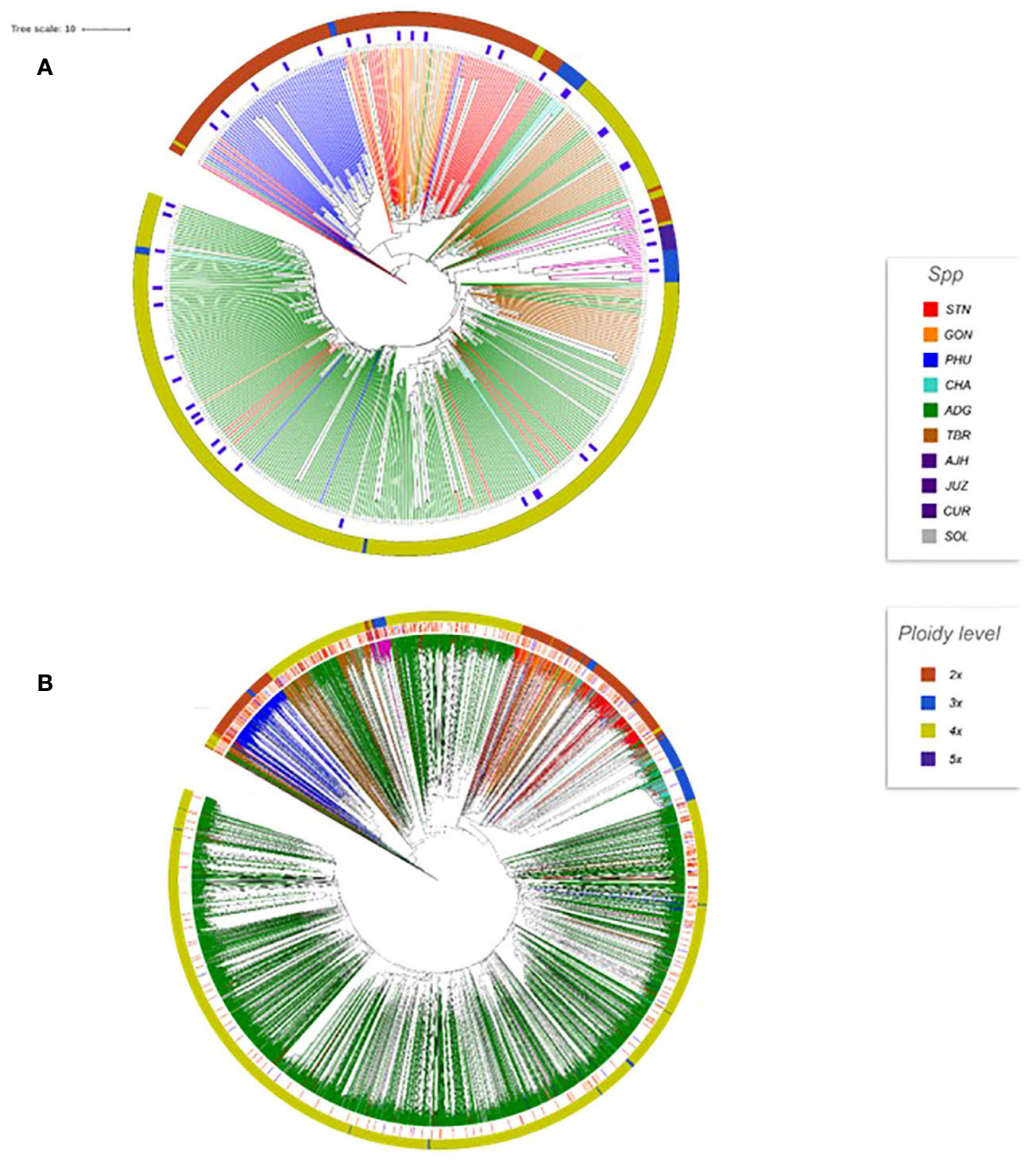
**(A)** Dendrogram of the 451 core and mini core accessions. The outer ring is the ploidy level. The branches are colored based on species designation. The blue rectangular blocks designate the selected mini core accessions (45 accs). **(B)** Dendrogram of the 3860 accessions with the core accessions marked as red lines (inside the ploidy circle) and the mini core marked with blue lines (inside the ploidy circle).

Although Core Hunter (ver 3.01) was the primary tool utilized to preselect the core subset, after selection by Core Hunter, the potato curator used agronomic traits and traditional potato knowledge to adjust a few of the selections based on over 30+ years of experience with potato and extensive knowledge on user preference resulting in a selection of 451 accessions for the CIP cultivated potato core collection. This core collection consists of 238 ADG, nine AJH, six CHA, seven CUR, 18 GON, seven JUZ, 55 PHU, 51 STN, 46 TBR, and 14 SOL accessions. A similar approach was then utilized to select 45 accessions from the 451 core accessions to develop the mini core collection to create a more manageable subset for research and trait identification (**Figure 9**). The mini core passport data along with pictures of the tubers can be seen and ordered through the CIP Genebank website https://genebank.cipotato.org/gringlobal/methodaccession.aspx. The mini core contains 19 ADG, two AJH, three CHA, two CUR, three GON, two JUZ, five PHU, five STN, and four TBR accessions. The dendrogram of the core and mini core can be seen in **Figure 9** in relation to the entire germplasm set, demonstrating good coverage relative to the entire germplasm collection with every cultivated species represented. The core and mini core collections at CIP have been requested for several research projects and have been successfully used to screen for novel sources of late blight, crop efficiencies via remote sensing, and to elucidate and reconstruct the origins of potato (Gutaker et al., 2019; Silva-Diaz et al., 2020; Perez et al., 2022). These subsets are available upon request to the CIP genebank.

## Conclusions

Genotyping germplasm collections is a major undertaking especially for large collections; however, the data produced from these projects are extremely valuable for the overall management of the germplasm and responding to potato researchers using the collection, especially breeders. Moreover, genotyping data in clonal collections can provide a framework and knowledge of the overall genetic integrity of accessions in a collection to be maintained by setting a baseline with fingerprinting data on each accession, which can be checked over the years to ensure no mistakes in manipulation and handling occur and to quickly rectify them when they do occur. Genotyping clonal accessions when they first are introduced into a collection can help facilitate integrity through monitoring of their fingerprints over time. When paired samples exist of accessions like in this study, genetic identity can be further checked by genotyping multiple samples of the same clonal accession to ensure that genetic identity is being maintained. Further, genotyping allowed a deeper understanding into the identity, genetic diversity, redundancy, relatedness, hybrid origin, and introgression of a germplasm collection that is difficult to grasp when just evaluating a subsection of a germplasm collection. The genotyping data provided new valuable information on inter- and intraspecific relationships and the hybrid origin of cultivated potato species and specific potato accessions. In this study, our data demonstrated that the level of introgression and hybridization of cultivated potato was high especially within ADG and CUR accessions. However, most all species and accessions contained quite a bit of admixture and demonstrated that potato species easily exchange alleles. Many individual genotypes were derived of alleles from two or more genetic clusters. Several potato species appeared to be hybrids such as GON and JUZ while other species had multiple introgressions (CUR, ADG). Further, this data supports both commonly used systems (Hawkes and Spooner) of potato taxonomy depending on the species in question and the analysis considered. For example, the lumping of species (Spooner) such as CHA and STN were supported in this data by a single lineage in STRUCTURE; however, PHU which Spooner lumped together and eliminated as a species, appeared to be a unique lineage which supports Hawkes’s taxonomy. The level of admixture for ADG would favor Spooner where a defined species level would be hard to ascertain. The data overall demonstrated the complexity and extensive hybridization that has occurred in the evolution of cultivated potato species and further how some species may be over described especially considering the amount of gene flow among the species. The STRUCTURE data also provided evidence for the putative species classification of accessions not previously classified that were new acquisitions to the genebank or material that was never able to be classified which is extremely valuable since potato taxonomists are a limiting factor. Further, genotyping of the CIP collection allowed for the first-time comparison of material conserved at other locations. A subsection of the Seed Savers potato collection was genotyped and compared to the 3860 potato samples at CIP and no overlap, or genetic redundancies were found. Future work may include working with other collections to genotype their material and rationalize collections. The SNP data and curator knowledge of the collection was further utilized to select a core and mini core collection to represent the majority of the genetic diversity in the genebank collection which can greatly help researchers measure traits that are expensive or unreasonable to measure collection wide. This is the first time a core or mini core collection has been developed from the potato genebank at CIP. The SNP data also allowed a confirmation of ploidy levels of the accessions since many were routinely classified based on species alone. Overall, genotyping has led to many insights on the diversity and population structure of one of the world’s largest cultivated potato germplasm collections.

## Data availability statement

The datasets presented in this study can be found in online repositories. The names of the repository/repositories and accession number(s) can be found below: https://data.cipotato.org/dataset.xhtml?persistentId=doi:10.21223/LBCFCF.

## Author contributions

NA: Investigation, Methodology, Project administration, Resources, Supervision, Visualization, Writing – original draft, Writing – review & editing. OC: Data curation, Formal analysis, Software, Validation, Visualization, Writing – review & editing. JS-T: Data curation, Formal analysis, Methodology, Software, Validation, Visualization, Writing – review & editing. RG: Writing – review & editing, Data curation, Methodology, Resources, Supervision, Validation. AP: Data curation, Methodology, Resources, Supervision, Writing – review & editing. RV: Methodology, Resources, Supervision, Writing – review & editing. MD: Data curation, Methodology, Resources, Visualization, Writing – review & editing. CM: Data curation, Resources, Supervision, Writing – review & editing. VA: Resources, Writing – review & editing. NM-C: Data curation, Formal analysis, Methodology, Resources, Software, Supervision, Writing – review & editing. PK: Investigation, Supervision, Writing – review & editing. JC: Data curation, Formal analysis, Software, Visualization, Writing – review & editing. DD: Data curation, Methodology, Resources, Writing – review & editing. DE: Conceptualization, Funding acquisition, Investigation, Project administration, Supervision, Writing – original draft.
